# Involvement of RBP-J interacting and tubulin-associated protein in the distribution of protein regulator of cytokinesis 1 in mitotic spindles

**DOI:** 10.3389/fcell.2024.1472340

**Published:** 2025-01-07

**Authors:** Julia Caspers, Andreas Ritter, Badi Bahrami, Samira Catharina Hoock, Susanne Roth, Alexandra Friemel, Franz Oswald, Frank Louwen, Nina-Naomi Kreis, Juping Yuan

**Affiliations:** ^1^ Obstetrics and Prenatal Medicine, Department of Gynecology and Obstetrics, University Hospital Frankfurt, J. W. Goethe-University, Frankfurt, Germany; ^2^ Center for Internal Medicine, Department of Internal Medicine I, University Medical Center Ulm, Ulm, Germany

**Keywords:** RITA, mitotic defects, cytokinesis failure, PRC1, the central spindle

## Abstract

The protein regulator of cytokinesis 1 (PRC1) is a key regulator of microtubule crosslinking and bundling, which is crucial for spindle formation and cytokinesis. RITA, the *R*BP-J *i*nteracting and *t*ubulin-*a*ssociated protein, is a microtubule associated protein. We have reported that RITA localizes to mitotic spindles modulating microtubule dynamics and stability as well as to spindle poles affecting the activity of Aurora A. As defective chromosome congression and segregation are the most remarkable features of cells depleted of RITA, we aimed to explore further potential related mechanisms, using various cellular and molecular techniques, including clustered regularly interspaced short palindromic repeats technique/deactivated CRISPR-associated protein 9 (CRISPR/dCas9), mass spectrometry, confocal microscopy, immunofluorescence, immunoprecipitation and Western blot analysis. Here, we show that FLAG-RITA precipitates PRC1 and tubulin, and that these two proteins co-localize in the central region of the central spindle. Reduction of RITA enlarges the staining area of PRC1 in mitotic spindles as well as in the central spindle. Its suppression reduces the inter-centromere distance in metaphase cells. Interestingly, microtubule bundles of the central spindle are often less organized in a non-parallel pattern, as evidenced by increased angles, relative to corresponding separating chromosomes. These data suggest a novel role for RITA in mitotic distribution of PRC1 and that its deregulation may contribute to defective chromosome movement during mitosis. As both RITA and PRC1 are closely associated with malignant progression, further work is required to elucidate the detailed molecular mechanisms by which RITA acts as a modulator in central spindle formation and cytokinesis.

## 1 Introduction

Microtubules (MTs) mediate various cellular activities, such as chromosome congression in metaphase and segregation in anaphase. During anaphase, the central spindle forms and elongates between the segregating chromosomes (Wadsworth, 2021). The central spindle is composed of antiparallel MTs and associates with numerous microtubule-associated proteins (MAPs), including protein regulator of cytokinesis (PRC1) and kinesin family member 4A (KIF4A), and is controlled by multiple regulators, such as Polo-like kinase 1 (Plk1), Aurora B and phosphatase PP2A (Holder et al., 2019; Wadsworth, 2021; Ali and Stukenberg, 2023; Kalous and Aleshkina, 2023). The central spindle generates forces that impact chromosome segregation and spindle elongation, determines the position of the contractile ring and results in the separation of two daughter cells (Wadsworth, 2021), which is the final step of cell division, termed cytokinesis. Cytokinesis ensures the equal distribution of genomic and cytoplasmic material between the two nascent daughter cells (D’Avino et al., 2015). Deregulated cytokinesis contributes to defective mitosis, polyploidy, and chromosomal instability (D’Avino et al., 2015; Lens and Medema, 2019), hallmarks of cancer (Hanahan and Weinberg, 2011).

RITA, the *R*BP-J *i*nteracting and *t*ubulin-*a*ssociated protein, is a member of the MAP family (Wacker et al., 2011; Steinhauser et al., 2017; Vicente and Wordeman, 2019). Interestingly, GFP-RITA localizes to MT-based structures, such as centrosomes, spindle poles, the mitotic spindle, the central spindle and the midbody (Wacker et al., 2011; Steinhauser et al., 2017). We have shown that RITA affects the stability and dynamics of mitotic MTs (Steinhauser et al., 2017) as well as the activity of Aurora A at spindle poles (Kreis et al., 2019), crucial for a faithful mitotic progression. Its depletion alters mitotic MT dynamics, activates Aurora A and causes severe mitotic defects (Steinhauser et al., 2017; Kreis et al., 2019). Interestingly, RITA has been reported to be overexpressed or downregulated in diverse primary tumor entities (Wang et al., 2013; Wang et al., 2014; Forbes et al., 2015), suggesting the importance of its proper regulation. Moreover, elevated RITA expression is associated with unfavorable clinical outcome in anal carcinoma treated with concomitant chemoradiotherapy (Rodel et al., 2018). In addition, RITA regulates cell migration and invasion of cancer cells by impacting the turnover of focal adhesions through its interference with the dynamics of MTs and actin filaments (Hoock et al., 2019). In line with this observation, RITA affects migration and invasion of trophoblastic cells (Wildner et al., 2019), which share many features with cancer cells (Louwen et al., 2012; Louwen et al., 2022). These findings point to the notion that RITA could be an important player in malignant progression.

As defects in chromosome congression and segregation are the most prominent features in RITA-depleted cells (Steinhauser et al., 2017; Kreis et al., 2019), we examined the underlying molecular mechanisms in more detail. Focusing on anaphase, we hypothesized a potential role of RITA in the central spindle organisation. In the present work, we investigated the novel involvement of RITA in the modulation of the central spindle by affecting the distribution of PRC1.

## 2 Materials and methods

### 2.1 Cell culture, preparation of mouse embryonic fibroblasts, transfection, viability assay, and cell cycle analysis

Cervical carcinoma HeLa, osteosarcoma U-2 OS and retinal pigment epithelial cells immortalized with human telomerase reverse transcriptase subunit (hTERT RPE-1) were cultured as described previously (Sanhaji et al., 2013; Kreis et al., 2019; Moon et al., 2021). The generation of RITA knockout mice (heterozygous RITA+/− and homozygous RITA−/−), mouse embryo fibroblast (MEF) isolation, genotyping and culture were previously described (Steinhauser et al., 2017).

The sequences of siRNA against the coding region of RITA and its 3′-untranslated region are GGA AGA AGA ACA AAU ACA G (siRITA #1) and AGG GAA CCC CAG GUA UUA AUU (siRITA #2) (Sigma-Aldrich), respectively. Control siRNA was obtained from Qiagen (Hilden). siRNAs (30 nM) were transiently transfected into cells with Oligofectamine^TM^ (Thermo Fisher Scientific, Dreieich), as reported (Kreis et al., 2016). Cloning of GFP- or FLAG-full length RITA was previously described (Wacker et al., 2011; Steinhauser et al., 2017). DNA was transfected as reported (Kreis et al., 2016).

Cell viability was assessed via CellTiter-Blue^®^ assay (#G808B, Promega GmbH, Walldorf), as instructed. For cell cycle evaluation, cells were harvested, washed with PBS, fixed with chilled 80% ethanol at 4°C for 30 min and were treated with 1 mg/mL of RNase A (#232-646-6, Sigma-Aldrich, Taufkirchen) and stained with 100 μg/mL of propidium iodide (PI) (#P1304MP, Sigma-Aldrich) at 37°C for 30 min. DNA content of about 10,000 cells was determined using a FACSCalibur^TM^ (BD Biosciences, Heidelberg), as reported (Kreis et al., 2015).

### 2.2 Generation of stable CRISPR/dCas9 cell lines

Following primers were used for the generation of RITA CRISPRa (activation) or CRISPRi (interference) plasmids: 1 µL (100 µM) of forward primers for CRISPRa: ttg​GGT​GTG​TAC​TAG​GCC​GCC​GAg​ttt​aag​agc, and for CRISPRi: ttg​GCG​AGC​CAA​GAT​GCT​CAG​GTg​ttt​aag​agc; and 1 µL (100 µM) of reverse primers for CRISPRa: tta​gct​ctt​aaa​cTC​GGC​GGC​CTA​GTA​CAC​ACC​caa​caa​g, and for CRISPRi tta​gct​ctt​aaa​cAC​CTG​AGC​ATC​TTG​GCT​CGC​caa​caa​g. 23 μL purified water and 25 µL annealing buffer [200 mM CH₃COOK, 60 mM HEPES-KOH pH 7.4, 4 mM Mg(CH₃COO)₂] were mixed with corresponding primers, followed by annealing at 95°C for 5 min and incubated at room temperature (RT) for 15 min. The annealed oligonucleotides were diluted at 1:10 with purified water. 1 μL (25 ng) of pCRISPRia-V2 (Addgene, Watertown), digested with *BstXI* and *BlpI*, was mixed with 0.5 µL annealed oligonucleotides (1:10 of CRISPRa/i), 0.5 µL T4 ligase, 0.5 µL T4 ligase buffer and 2.5 µL purified water, and incubated at RT for 30 min. The mixtures were transformed into XL-1 blue competent cells, plated on ampicillin agar plates and incubated at 37°C for overnight. Clones were picked and DNA was isolated (EXTRACT ME^®^ Plasmid Mini Kit, Qiagen) for sequencing (Eurofins Genomics Support, Constance). The CRISPR plasmids were then amplified for transfection into HEK293T cells.

Establishment of HeLa and hTERT RPE-1 CRISPR/dCas9 sgRITA cell lines: lentiviral particles were generated by transfecting HEK293T cells with 12.5 µg of the packaging vector PsPax2, 4 µg of envelope vector VSV-G and 7.5 µg of sgRNA of the insert of interest (CRISPRa- or CRISPRi, respectively), via calcium chloride (CaCl_2_) precipitation. Transfected HEK293T cells were incubated for 48 h and supernatants containing viral particles were harvested and filtered through a 0.45 µm filter. The particles (5 mL supernatant) were added to HeLa CRISPRi or hTERT RPE-1 CRISPRi/a cell lines (gifts from Dr. Jonathan Weissman), or HeLa CRISPRa cell line (GeneCopoeiaTM, Rockville). The transduction was repeated after 24 h and transduced cells were selected with puromycin (Invivogen, San Diego) for about 2 weeks (four to five passages) to obtain cell lines stably expressing the gene of interest (GOI).

### 2.3 RNA extraction and real-time PCR (RT-PCR) analysis

For RT-PCR analysis, total RNAs were extracted with EXTRACT ME TOTAL RNA Kit (#EM09.2.-250, 7Bioscience, Neuenburg). Reverse transcription was performed using GoScript^TM^ Reverse Transcription Mix and Random Primers (#A2801, Promega, Madison), as instructed. The primers and probes for *GAPDH* (Hs02758991_g1) and *RITA* (Hs03044851_m1) were obtained from Thermo Fisher Scientific (Dreieich). Real-time PCR was performed with a StepOnePlus Real-time PCR System (Applied Biosystems). The data were analyzed using StepOne Software v.2.3 (Applied Biosystems) and the results were shown as fold.

### 2.4 Western blot analysis and immunoprecipitation

For Western blot analysis, cells were lysed with RIPA buffer and the analysis was performed, as described (Steinhauser et al., 2017). The following primary antibodies were used for analyses: mouse monoclonal anti-α-tubulin, mouse monoclonal anti-acetylated tubulin antibody and mouse monoclonal anti-FLAG (#T6047, #T6793, #F1804, Sigma-Aldrich), mouse monoclonal as well as rabbit polyclonal anti-PRC1 (#sc-376983, #sc-8356, Santa Cruz Biotechnology, Heidelberg), and mouse monoclonal anti-GAPDH antibody (#GT239, GeneTex). The RITA antibody was designed and commercially produced (rabbit monoclonal IgG, Epitomics, Burlingame), as described (Steinhauser et al., 2017). Quantification of Western blot analysis was performed with ImageJ (National Institutes of Health).

For performing immunoprecipitation, HEK293T cells were transiently transfected with FLAG vector or FLAG-RITA for 48 h and lysed with lysis buffer [20 mM TRIS, pH 8.2, 150 mM NaCl, 1% Triton-X-100, phosphatase and protease inhibitor cocktail (Roche, Mannheim)]. To examine if MT-interfering agents could affect the interaction, transfected cells were further treated with DMSO, 10 µM of paclitaxel (NeoTaxan^®^, Hexal AG) or 10 µM of nocodazole (Sigma-Aldrich) for 30 min at 37°C, which were reported to effectively stabilize or destabilize MTs, respectively (Shannon et al., 2005; Smyth et al., 2007). Cells were then lysed with RIPA buffer containing DMSO, nocodazole or paclitaxel to further destabilize or stabilize MTs. Mouse monoclonal anti-α-tubulin antibody (#T6047, Sigma-Aldrich) and 20 µL Protein G Sepharose^TM^ 4 Fast Flow beads (GE Healthcare, Uppsala) or anti-FLAG agarose gel beads (#A2220-1ML, Sigma-Aldrich) were added and incubated on a rotator at 4°C for overnight. The beads were washed three times for further SDS-PAGE.

### 2.5 Indirect immunofluorescence staining, microscopy, intensity/area evaluation and measurement of inter-centromere distance

Indirect immunofluorescence staining was performed, as described (Ritter et al., 2016). Following primary antibodies were used: rabbit polyclonal anti-α tubulin antibody (#ab15246, Abcam, Cambridge), human ACA (human anti-centromere antibody, ImmunoVision, Springdale, AR), mouse monoclonal anti-PRC1 (#sc-376983, Santa Cruz Biotechnology), rabbit polyclonal anti-PRC1 (#sc-8356), mouse monoclonal anti-α-tubulin (#F2168, Sigma Aldrich), rabbit polyclonal anti-RITA antibody (Atlas Antibodies, Stockholm), rabbit polyclonal antibody against pericentrin (#ab28144, Abcam), and mouse monoclonal antibodies against FLAG-tag (#F3165, Sigma-Aldrich). FITC-, Cy3- and Cy5-conjugated secondary antibodies were obtained from Jackson Immunoresearch (Newmarket). DNA was stained using DAPI (4′,6-diamidino-2-phenylindole-dihydrochloride) (Roche).

Cells were evaluated using an Axio Imager 7.1 microscope (Zeiss, Göttingen) and images were taken using an AxioCam MRm camera (Zeiss). Cells were also imaged using a confocal laser scanning microscope (CLSM) (Leica CTR 6500) with an HCXPI APO CS 63.0 × 1.4 oil objective and the LAS AF software (Leica). A series of Z-stack images (4-fold zoom) were captured at 0.5 µm intervals. For quantitative measurement of mean fluorescence intensity (MFI) or area, fluorescence intensity or area from a standard region of interest (ROI) was integrated using the AxioVision software (Zeiss) and background intensity or area (standard area outside ROI) was subtracted. All images in each experiment were taken with the same wave intensity and exposure time. All experiments were independently performed at least three times unless otherwise specified.

For measuring the inter-centromere distance, cells were stained for the MT marker α-tubulin, the centromere marker ACA and DNA (DAPI). The distance was measured by using a confocal laser scanning microscope (CLSM, Leica CTR 6500) with LAS AF software (Leica, Heidelberg) and images were processed using Adobe Photoshop, as reported (Ritter et al., 2015).

### 2.6 Calculation of Pearson correlation coefficient

The Pearson correlation coefficient between RITA and PRC1 was calculated in the following cell lines: HeLa CRISPRa cells stably expressing RITA and stained for endogenous RITA, PRC1 and DNA; HeLa cells transiently transfected with GFP-RITA and stained for PRC1 and DNA; and HeLa CRISPRi sgRITA cells transiently transfected with FLAG-RITA and stained for FLAG, PRC1 and DNA. The immunofluorescence signals of RITA/tagged RITA and PRC1 in mitotic spindles, the central spindle, the midzone and the midbody were measured by microscopy for calculating the Pearson correlation coefficient as instructed (Dunn et al., 2011). Co-localization analyses were performed using NIH ImageJ Plugin JACop (Bolte and Cordelieres, 2006). Pearson correlation coefficient is interpreted as following: a value of +1 is defined as a positive linear relationship, −1 as a negative one and 0 as no linear relationship.

### 2.7 Angle measurement of PRC1 stained MT bundles

Cells were stained for PRC1 and ACA. Fluorescence images of early anaphase cells were taken with an AxioCam MRm camera (Zeiss). The resulting signals were analyzed using the integrated angle tool in ImageJ. Centerlines of associated chromosomes marked by ACA staining were generated. The relative angle (θ) between the centerlines and the PRC1 stained MT bundles was quantified for each individual bundle, with modification as reported (Carlini et al., 2022). The angle formed by this line with the chromosome-chromosome axis (paired ACA staining) was calculated. Angles above 40° were excluded, because of the possibility that these MT bundles were incorrectly attached to wrong chromosomes. Representative images were generated with a confocal laser scanning microscope (CLSM) using Z-stack images with an HCXPI APO CS 63.0 × 1.4 oil objective (Leica CTR 6500, Heidelberg, Germany). A series of Z-stack images were captured at 0.5 μm intervals for overlays. Representatives are generated by superimposing (overlay) individual images from confocal Z-sections.

### 2.8 Statistical analysis

Outliers were identified using the Grubbs’ test (GraphPath QuickCalcs, San Diego). The normality of data distribution was analyzed with the Shapiro-Wilk test. Statistical significance was assessed with the Student’s t-test, or, if not Gaussian distributed, with the Mann-Whitney U test, unless otherwise described. A difference was defined as statistically significant if *p* < 0.05.

## 3 Results

### 3.1 Depletion of RITA increases defective mitosis and cytokinesis

**FIGURE 1:**
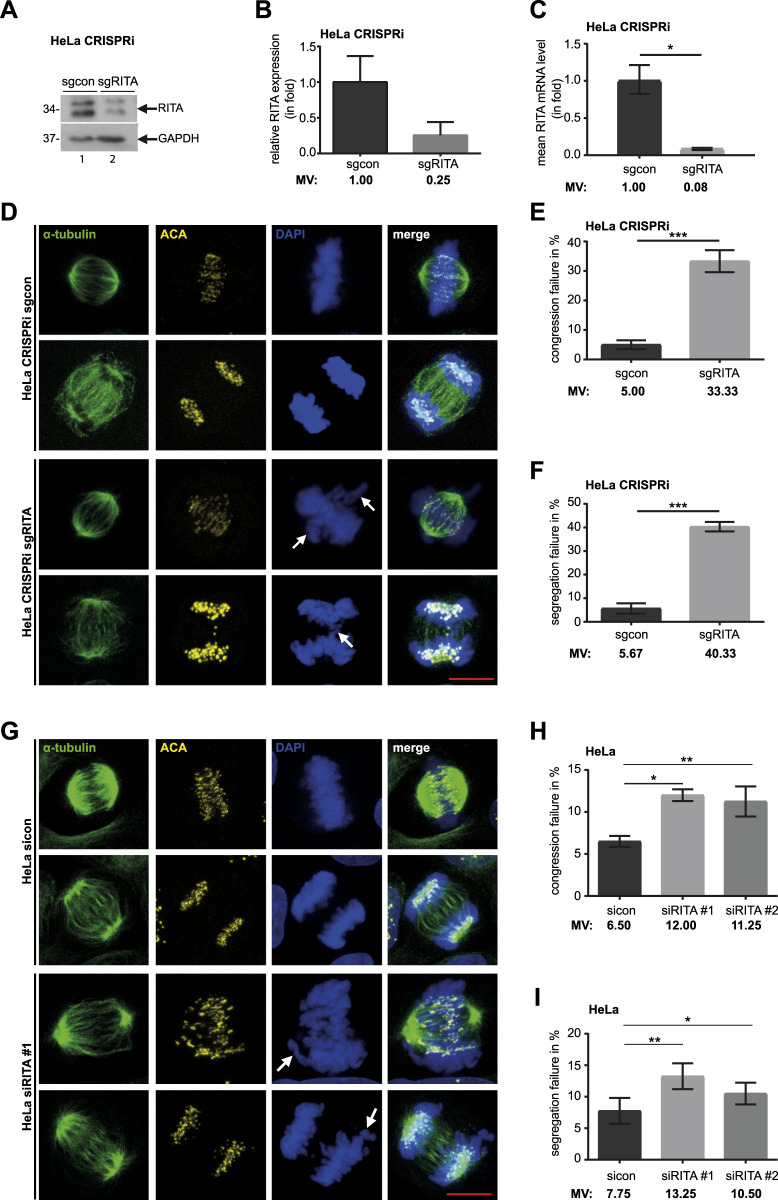
Knockdown of RITA results in mitotic defects. **(A)** Cellular lysates from HeLa CRISPRi sgRITA and control cells were prepared for Western blot analysis with RITA antibody. GAPDH served as loading control. **(B)** The average protein levels of RITA normalized to the loading control in HeLa CRISPRi cells. The results are from three independent experiments. **(C)** Total RNAs were extracted for RT-PCR and gene levels of RITA are shown for HeLa CRISPRi sgcon and sgRITA cells. The results are from three individual experiments. **(D)** HeLa CRISPRi sgRITA cells and control HeLa CRISPRi sgcon cells were stained for the MT marker α-tubulin (green), the kinetochore/centromere marker ACA (yellow) and DNA (DAPI, blue) for microscopy. Representative confocal images are shown. White arrows indicate defects in chromosome alignment in metaphase and chromosome segregation in anaphase. Scale: 10 µm. **(E, F)** Chromosome congression **(E)** and segregation defects **(F)** were evaluated in HeLa CRISPRi sgRITA and HeLa CRISPRi sgcon cells. Three individual experiments were performed (n = 3, 100 mitotic cells for each condition in each experiment) and the results are shown as mean ± SEM. **(G)** HeLa cells were transiently transfected with siRNA #1 targeting the coding region of RITA or siRNA #2 targeting its 3′-untranslated region for 48 h and stained as indicated. Representative confocal images are shown. White arrows depict failures in chromosome congression and segregation. Scale: 10 µm. **(H, I)** Evaluation of misaligned chromosomes **(H)** and failed segregation **(I)** in HeLa cells transiently depleted of RITA and control cells. The results are from four independent experiments (n = 4, 100 mitotic cells for each condition in each experiment) and presented as mean ± SEM. Student’s t-test was used. *p < 0.05, **p < 0.01, ***p < 0.001.

To further substantiate the mitotic phenotype observed in previous studies (Steinhauser et al., 2017; Kreis et al., 2019), we established stable RITA knockdown cervical carcinoma HeLa and retinal pigment epithelial hTERT RPE-1 cell lines, based on CRISPR/dCas9 technique (Jost et al., 2017; Moon et al., 2021), referred to hereafter as HeLa CRISPRi sgRITA and hTERT RPE-1 CRISPRi sgRITA cells, respectively. Compared to control HeLa CRISPRi sgcon cells, protein and mRNA levels of RITA were reduced in HeLa CRISPRi sgRITA cells (Figures 1A–C). Cell viability and cell cycle distribution were comparable between HeLa CRISPRi sgRITA cells and control cells (Supplementary Figure S1A, left and middle panels). Notably, the level of acetylated tubulin was elevated in HeLa CRISPRi sgRITA cells (Supplementary Figure S1A, right panel), as reported (Steinhauser et al., 2017), reinforcing the notion that RITA is involved in the modulation of MT stability.

To further analyze the mitotic phenotypes, HeLa CRISPRi sgRITA cells and their control HeLa CRISPRi sgcon cells were stained for the MT marker α-tubulin, the centromere marker anti-centromere antibody (ACA) and DNA, and mitotic defects were evaluated by microscopy (Figure 1D). Relative to control HeLa CRISPRi sgcon cells, HeLa CRISPRi sgRITA cells displayed more than six-fold defective chromosome alignment (5.00% vs. 33.33%, Figure 1E) and more than seven-fold abnormal chromosome segregation (5.67% vs. 40.33%, Figure 1F), including anaphase bridges and lagging chromosomes. This was further supported by HeLa cells transiently depleted of RITA with siRNA #1 targeting its coding region or siRNA #2 against its 3-untranslated region (Supplementary Figure S1B, right panel) showing obvious mitotic chromosome defects (Figures 1G–I). HeLa cells transiently depleted of RITA also demonstrated comparable cell viability and cell cycle profiles, as well as an increase in acetylated tubulin, compared to cells treated with control siRNA (sicon) (Supplementary Figure S1B). Furthermore, mitotic defects were also observed in hTERT RPE-1 CRISPRi sgRITA (Supplementary Figures S2A–C) as well as in hTERT RPE-1 cells transiently depleted of RITA (Supplementary Figures S2D–F). Both hTERT RPE-1 CRISPRi cells and hTERT RPE-1 cells transiently depleted of RITA displayed hardly changes in cell viability and cell cycle distribution but showed elevated acetylated tubulin levels compared to their respective control cells (Supplementary Figures S1C, D). In addition, failures in chromosome alignment and segregation were detectable in osteosarcoma U-2 OS cells transiently depleted of RITA (Supplementary Figure S3). Overall, these results substantiate the notion that RITA is required for successful mitosis and its depletion results in severe defects in chromosome movement during metaphase as well as anaphase.

**FIGURE 2:**
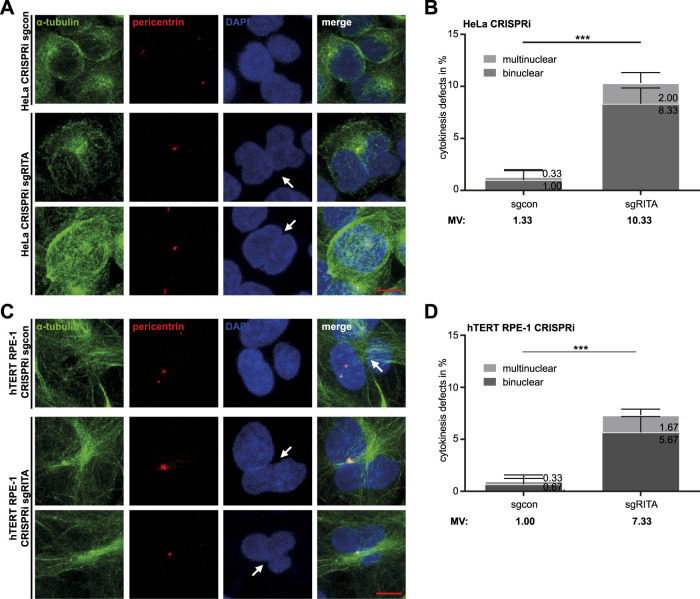
Defective cytokinesis in HeLa cells stably depleted of RITA. **(A)** HeLa CRISPRi sgRITA and HeLa CRISPRi sgcon cells were stained for pericentrin (red), α-tubulin (green) and DNA (DAPI, blue) for microscopy. Representative images are shown. White arrows indicate binuclear or multinuclear cells due to cytokinesis defects in HeLa CRISPRi sgRITA cells. Scale: 10 µm. **(B)** Evaluation of binuclear and multinuclear HeLa cells stably depleted of RITA. The results are from three independent experiments (n = 3, 100 mitotic cells for each condition in each experiment) and presented as mean ± SD. **(C)** hTERT RPE-1 CRISPRi sgRITA cells and their control cells were stained as indicated for microscopy. Representative images are shown. White arrows depict binuclear or multinuclear cells. Scale: 10 µm. **(D)** Evaluation of binuclear and multinuclear cells in hTERT RPE-1 CRISPRi sgRITA cells stably depleted of RITA and their control cells. The results are from three independent experiments (n = 3, 100 mitotic cells for each condition in each experiment) and presented as mean ± SD. Student’s t-test was used. ***p < 0.001.

As defective chromosome segregation is often associated with failed cytokinesis (Mierzwa and Gerlich, 2014; Wadsworth, 2021), we examined whether cytokinesis was properly carried out in CRISPRi cells. As expected, cytokinesis defects, including bi- and multinucleated cells, increased more than seven-fold in HeLa CRISPRi sgRITA cells compared to their corresponding control cells (Figures 2A, B). hTERT RPE-1 CRISPRi sgRITA cells displayed similar defects in cytokinesis (Figures 2C, D). These data indicate that RITA may also be required for cytokinesis.

### 3.2 FLAG-RITA precipitates with PRC1 and tubulin

**FIGURE 3:**
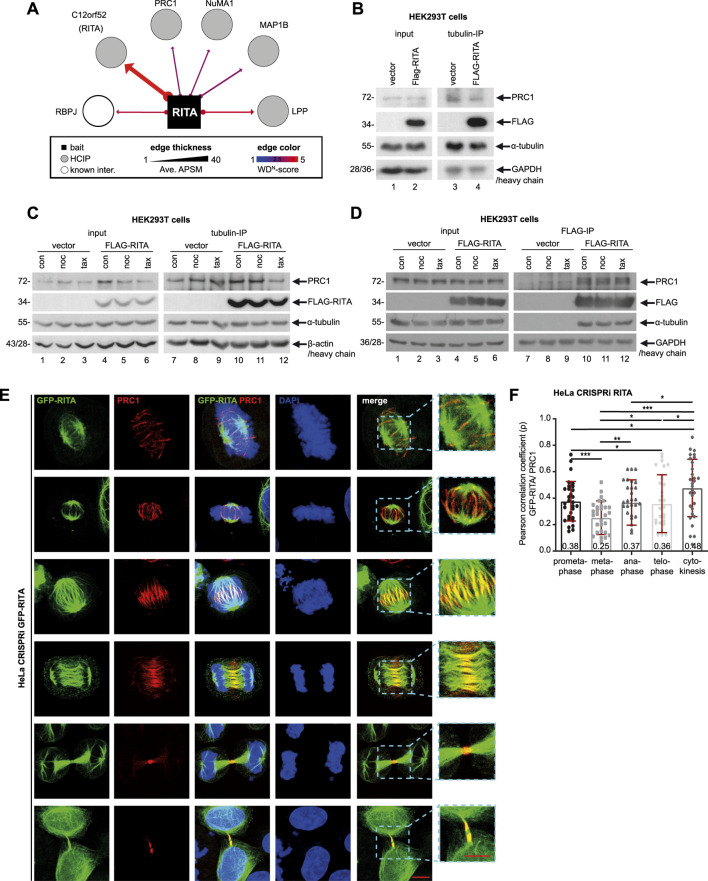
FLAG-RITA precipitates PRC1 and tubulin. **(A)** Mass spectrometry re-analysis of HA-tagged RITA immunoprecipitates from HEK293T cells revealed potential interaction partners of RITA. Potential partners with a weighted D score (WD score) ≥ 1 are presented. RBP-J, recombination signal binding protein for immunoglobulin kappa J region; PRC1, protein regulator of cytokinesis 1; NuMA, nuclear mitotic apparatus; MAP1B, microtubule-associated protein 1B; LPP, lipoma-preferred partner. **(B)** HEK293T cells were transiently transfected with FLAG-empty vector or FLAG-RITA plasmids for 48 h and cellular lysates were prepared for immunoprecipitation with tubulin antibody. Precipitates were analyzed by Western blot analysis with indicated antibodies. Left panel: input control. GAPDH served as loading control. Right panel: precipitates were analyzed via Western blot using antibodies as indicated. Antibody light chain was used as reference for precipitate loading. **(C)** HEK293T cells, transiently transfected as in **(B)** for 48 h, were treated with DMSO, 10 µM of nocodazole or 10 µM of paclitaxel for 30 min. Cellular lysates were prepared for immunoprecipitation with tubulin antibody. Precipitates were analyzed by Western blot analysis with indicated antibodies. Left panel: input control. β-actin served as loading control. Right panel: precipitates were analyzed via Western blot using indicated antibodies. Antibody heavy chain was used as reference for precipitate loading. **(D)** HEK293T cells were treated as in **(C)** and cellular lysates were prepared for immunoprecipitation with anti-FLAG antibody. Precipitates were analyzed by Western blot analysis with indicated antibodies. Left panel: input control. GAPDH served as loading control. Right panel: precipitates were analyzed via Western blot using indicated antibodies. Antibody heavy chain was used as reference for precipitate loading. **(E)** Subcellular localization of GFP-RITA and PRC1. HeLa CRISPRi sgRITA cells were transiently transfected with GFP-RITA for 48 h and cells were stained for PRC1 (red) and DNA (DAPI, blue) for microscopy. Representative confocal images are shown. Scale: 10 µm. Insets: magnified regions. Inset scale: 10 µm. **(F)** GFP-RITA transfected HeLa CRISPRi cells were stained for PRC1 (red) and DNA (DAPI, blue) and the co-localization of both proteins in mitotic spindles, the central spindle and the midbody was evaluated via Pearson correlation coefficient, where +1 equates to total positive linear correlation. The Pearson correlation coefficient is indicated as numbers at the column bottom. The correlation assay was independently performed three times (n = 3, 10 for each condition in each experiment) and the results are presented as bar scatter blots, mean ± SD. Student’s t-test was used. *p < 0.05, **p < 0.01, ***p < 0.001.

The central spindle is essential for appropriate anaphase progression as well as accurate initiation and completion of cytokinesis (Wadsworth, 2021). As RITA coats MTs of the central spindle (Steinhauser et al., 2017) and its reduction resulted in defective chromosome segregation and failed cytokinesis, we hypothesized an involvement of RITA in the organization of the central spindle. To explore the potential interaction partners of RITA in the central spindle, we re-analyzed the data from a previous mass spectrometry analysis performed with HEK293T cells transiently transfected with HA-tagged RITA (Hoock et al., 2019). In addition to known RITA partners, such as RBP-J (recombination signal binding protein for immunoglobulin kappa J region) (Wacker et al., 2011) and LPP (lipoma-preferred partner) (Hoock et al., 2019), PRC1, NUMA1 (nuclear mitotic apparatus protein 1) and MAP1B (microtubule-associated protein 1B) were among the RITA interaction partners (Figure 3A). PRC1 immediately attracted our attention, as it is an important MT-bundling protein and a key regulator of the central spindle assembly (Li et al., 2018; She et al., 2019).

Since various subtypes of tubulin are richly present in the mass spectrometry data and RITA directly interacts with tubulin (Steinhauser et al., 2017), we asked if tubulin could be involved in mediating RITA’s association with PRC1. HEK293T cells were transiently transfected with FLAG-RITA or FLAG empty vector for 48 h and cellular lysates were prepared for immunoprecipitation with tubulin antibody. The precipitates were analyzed via Western blot using PRC1 and FLAG antibodies. Indeed, tubulin precipitated both PRC1 and FLAG-RITA from the lysates of HEK293T cells transfected with FLAG-RITA plasmids, whereas it only interacted with PRC1 from the lysates of HEK293T cells transfected with FLAG empty vector (Figure 3B). To examine if this association is affected by MT-interfering agents, transfected HEK293T cells were treated with DMSO, 10 µM of nocodazole, or 10 µM of paclitaxel for 30 min, which were reported to effectively destabilize (Smyth et al., 2007) or stabilize intracellular MTs (Shannon et al., 2005). Cellular lysates were prepared for immunoprecipitations. While PRC1 in the precipitate was hardly changed upon a short treatment of nocodazole, its level was slightly reduced after a short treatment of MT stabilizer paclitaxel (Figure 3C), suggesting that quickly stabilized MTs might impair the precipitation of tubulin with PRC1. Moreover, to study if FLAG-RITA could physically interact with PRC1 and whether the interaction is affected by MT drugs, cellular lysates were also prepared for immunoprecipitation with anti-FLAG antibody. FLAG-RITA precipitated both PRC1 and tubulin (Figure 3D). *In vitro* binding assays using purified RITA and PRC1 proteins are needed to examine the possibility of a direct interaction between these two proteins. In addition, a short treatment of MT drugs hardly changed the PRC1 amounts in the precipitates (Figure 3D), indicating that a short time intervention of MT stability is inefficient to alter the association, at least in non-synchronized HEK293T cells overexpressing RITA. Further investigations, including time and dosage kinetics of MT-interfering agents, using other cell lines, lysates from different cell cycle phases or MT extracts alone, are required to answer the questions, if/when/how the MT drugs interfere with the interaction.

We then examined the subcellular localization of RITA and PRC1 throughout mitosis. To exclude potential effects of endogenous RITA, HeLa CRISPRi cells, in which RITA expression was suppressed (Figures 1A, B; Supplementary Figure S1A, right panel), were used. These cells were transiently transfected with GFP-RITA for microscopy. While slight but clear co-localization of RITA and PRC1 was detectable along mitotic spindle MTs in prometa- and metaphase (Figure 3E, 1^st^ and 2^nd^ panels), the co-localization was also found in early and late anaphase (Figure 3E, 3^rd^ to 4^th^ panels). Specifically, PRC1 accumulated in the middle region of the central spindle, whereas RITA was distributed over the spindle and overlapped with PRC1 in the center of the central spindle (Figure 3E, 3^rd^ and 4^th^ panels). RITA continued its escort for PRC1 in the midbody during cytokinesis (Figure 3E, 5^th^ and last panels). To further determine the subcellular relationship between these two proteins, their co-localization in mitotic spindles, the central spindle and the midbody was evaluated using the Pearson correlation coefficient, where +1 equates to total positive linear correlation. Interestingly, though the coefficients were not high, the co-localization correlation of GFP-RITA and PRC1 was detectable in mitotic spindles of prometaphase and metaphase, the central spindle of anaphase, the midzone of telophase and the midbody of cytokinesis (Figure 3F), indicating that portions of these two proteins co-localize throughout mitosis and cytokinesis.

To underline this observation, HeLa CRISPRa sgcon and HeLa CRISPRa sgRITA cells with enhanced RITA were generated (Supplementary Figures S6A–C). These cells were stained for endogenous RITA and PRC1, and similar co-localization of these two proteins was also observed in anaphase, although RITA staining was weak due to the low sensitivity of the RITA antibody (Supplementary Figure S4A). Furthermore, HeLa CRISPRi cells were transiently transfected with FLAG-RITA (Supplementary Figure S4D) and stained for microscopy. Again, RITA was found over the entire central spindle, whereas PRC1 was concentrated on the middle part of the central spindle and overlapped with RITA (Supplementary Figure S4C). The co-localization correlation coefficient of RITA/FLAG-RITA and PRC1 was relatively high in the central spindle of anaphase cells and the midbody of cytokinesis (Supplementary Figures S4B, E). These results indicate that RITA may interact with PRC1 throughout mitosis, possibly indirectly through tubulin, and that these two proteins particularly co-localize in the middle region of the central spindle in anaphase and the midbody during cytokinesis.

### 3.3 Depletion of RITA enlarges the staining area of PRC1 in mitotic spindles and decreases the inter-centromere distance of sister chromatids

**FIGURE 4:**
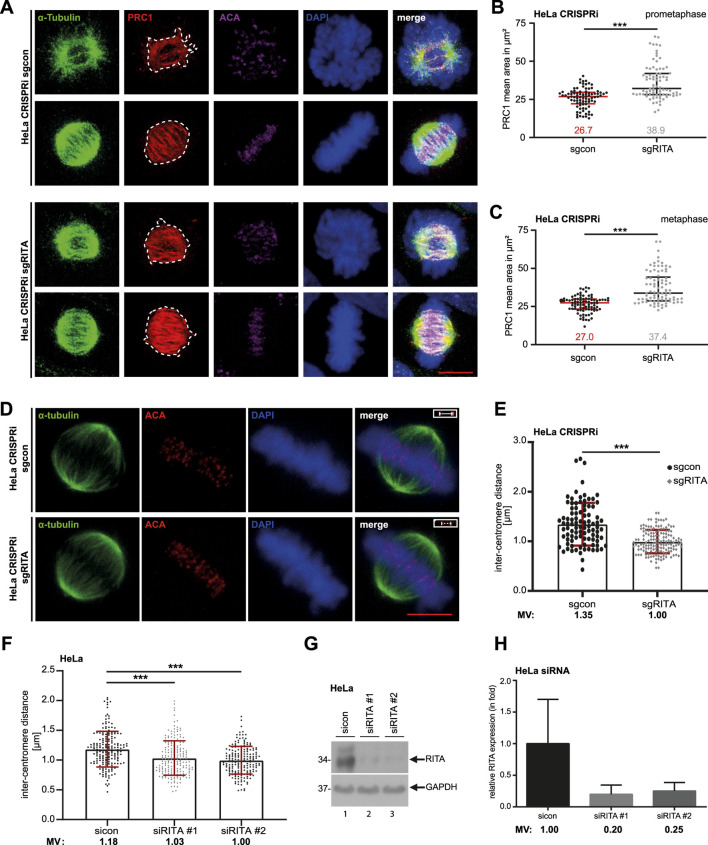
Reduced RITA enlarges the PRC1 staining area in mitotic spindles and is associated with decreased inter-centromere distance. **(A)** HeLa CRISPRi sgRITA and their control cells were stained for α-tubulin (green), PRC1 (red) and DNA (DAPI, blue) for microscopy. Representative confocal images of prometaphase and metaphase cells are shown. Scale: 7.5 µm. **(B, C)** The staining area of PRC1 was evaluated in the mitotic spindle in prometaphase **(B)** and metaphase **(C)** (n = 3, 30 cells for each condition in each experiment). The results are presented as scatter plots with mean ± SD. Mann-Whitney U test was performed. ***p < 0.001. **(D, E)** HeLa CRISPRi sgRITA and control cells were stained for ACA, α-tubulin and DNA. The inter-centromere distance was measured using the LAS AF software (n = 3, 94–149 pairs for each condition and each experiment). Representative cells are shown **(D)**. Scale bar: 7.5 μm. Insets: paired centromeres. The results are presented as bar scatter plots, mean ± SD **(E)**. Mann-Whitney U test was performed. ***p < 0.001. **(F–H)** HeLa cells, transiently depleted of RITA with siRNA#1 or #2 for 48 h, were stained as indicated in **(D)**. **(F)** The inter-centromere distance was evaluated (n = 3, 188–197 pairs for each condition and each experiment) and the results are presented as bar scatter plots, mean ± SD. Mann-Whitney U test was performed. ***p < 0.001. **(G)** Cellular lysates from treated HeLa cells were prepared for Western blot analysis with RITA antibody. GAPDH served as a loading control. **(H)** The average protein levels of RITA normalized to the loading control in HeLa cells treated with siRNAs. The results are from three independent experiments.

PRC1 has been reported to be localized on MTs of mitotic spindles in a highly dynamic manner upon the start of mitosis (Jiang et al., 1998). Indeed, PRC1 was detectable in mitotic spindles (Figure 3D, 1^st^ and 2^nd^ panels). We were interested in whether RITA affected PRC1’s recruitment to MTs of mitotic spindles in prometaphase and metaphase cells. HeLa CRISPRi cells were stained for α-tubulin, PRC1, ACA and DNA for microscopic analysis (Figure 4A). The evaluation showed that the PRC1 staining area was significantly enhanced in mitotic spindles in prometaphase as well as in metaphase cells (Figures 4B, C). Given that RITA coats MTs and affects their features (Steinhauser et al., 2017), these data imply that RITA is required for PRC1’s proper distribution on the MTs of mitotic spindles.

Moreover, an appropriate tension between sister kinetochores/centromeres, frequently measured as the inter-centromere distance, is important for correcting mal-attachments between MTs and kinetochores (Andrews et al., 2004; Ritter et al., 2015). PRC1 has been reported to be localized in the bridging fibers that withstand the tension between sister-kinetochores in metaphase (Kajtez et al., 2016). To examine if depletion of RITA affects the inter centromere distance, HeLa CRISPRi sgRITA and control cells were stained for the centromere marker ACA and the MT marker α-tubulin for the evaluation via confocal microscopy (Figure 4D). Interestingly, the distance was significantly reduced in HeLa CRISPRi sgRITA cells compared to control cells (Figure 4E). Similar results were also obtained with HeLa cells transiently knocked down of RITA (Figures 4F–H). Reduced sister kinetochore/centromere distance could be attributed to the fact that RITA modulates MT stability/dynamics (Steinhauser et al., 2017) and the activity of Aurora A kinase (Kreis et al., 2019) that are crucial for a proper regulation of kinetochore-MT attachment dynamics (Maiato et al., 2004; DeLuca, 2017; DeLuca et al., 2018). These data indicate that RITA is involved in maintaining tension between sister-kinetochores and therefore in correcting mal-attachments between MTs and kinetochores.

### 3.4 Enlarged staining areas of PRC1 in the central spindle in the absence of RITA

**FIGURE 5:**
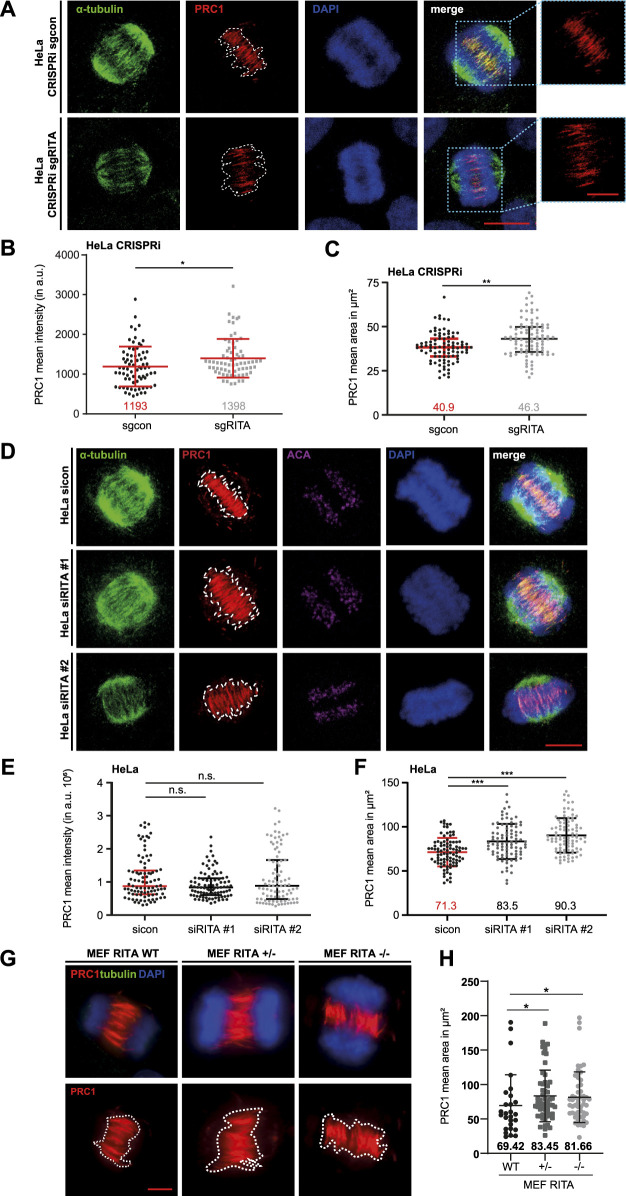
Enlarged area of PRC1 staining in the central spindle. **(A)** HeLa CRISPRi sgRITA and control cells were stained for α-tubulin (green), PRC1 (red) and DNA (DAPI, blue) for microscopy. Representative confocal images are shown. White dotted lines indicate the measured areas of PRC1 staining in the central spindle. Scale: 7.5 µm. Insets show magnified images of the PRC1 staining. Inset scale: 7.5 µm. **(B)** The staining intensity of PRC1 in the central spindle of early anaphase cells was evaluated (n = 3, 30 cells for each condition in each experiment). The results are presented as scatter plots with mean ± SD. **(C)** The staining area of PRC1 in the central spindle of early anaphase cells was evaluated (n = 3, 30 cells for each condition in each experiment). The results are presented as scatter plots with mean ± SD. **(D)** HeLa cells were transiently transfected with siRNA (sicon, siRITA#1 or siRITA#2) for 48 h and stained as indicated for microscopy. Representative confocal images are shown. White dotted lines indicate the measured areas. Scale: 10 µm. **(E)** The staining intensity of PRC1 was evaluated in the central spindle of early anaphase cells (n = 3, 30 cells for each condition in each experiment). The results are presented as scatter plots with mean ± SD. **(F)** The staining area of PRC1 in the central spindle of early anaphase cells was evaluated (n = 3, 30 cells for each condition in each experiment). The results are shown as scatter plots with mean ± SD. **(G)** Mouse embryonic fibroblasts (MEFs, wild type (WT), heterozygous RITA +/− and homozygous RITA −/−) were stained as indicated for microscopy. Representative images are shown. Scale: 5 µm. White dotted lines depict the measured areas. **(H)** The staining area of PRC1 was evaluated in the central spindle of MEFs. The results are based on two independent experiments (n = 2, 27 cells of RITA WT, 56 of RITA+/− and 53 of RITA−/−) and presented as scatter plots with mean ± SD. Mann-Whitney U test was used. *p < 0.05, **p < 0.01, ***p < 0.001.

We next focused on the functional relationship between RITA and PRC1 in the central spindle of anaphase cells. HeLa CRISPRi cells were stained for α-tubulin, PRC1 and DNA (Figure 5A). The signal intensity and the area of PRC1 staining in the central spindle in early anaphase cells were examined by microscopy. The evaluation revealed that the intensity of PRC1 staining was increased in the central spindle (Figure 5B). Like in mitotic spindles (Figures 4B, C), the staining area of PRC1 in the central spindle was also enlarged (Figure 5C). Moreover, while control cells demonstrated properly aligned MT bundles in the middle region of the central spindle (Figure 5A, upper panel), cells depleted of RITA often displayed less organized and non-parallel MT bundles (Figure 5A, lower panel).

To corroborate these observations, HeLa cells were transiently depleted of RITA with two different siRNAs targeting RITA (Supplementary Figure S1B, right panel) and stained as indicated for microscopic analysis (Figure 5D). Intriguingly, we could not observe an increase in the intensity of PRC1 staining in the central spindle of early anaphase cells (Figure 5E). While HeLa cells treated with siRNA #1 targeting the coding region of RITA enlarged the PRC1 staining area by 17%, siRNA #2 against its 3′-untranslated region had an increased area by 27% in the central spindle of early anaphase cells (Figure 5F). An enlarged area of PRC1 staining was also observed in the central spindle of hTERT RPE-1 CRISPRi sgRITA anaphase cells (Supplementary Figures S5A, B) as well as in hTERT RPE-1 and in U-2 OS cells transiently depleted of RITA (Supplementary Figures S5C–F).

Finally, the staining area of PRC1 in the central spindle was also enhanced in MEF RITA +/− and MEF RITA −/− cells, relative to wild type MEFs (Figures 5G, H). These results suggest an involvement of RITA in the proper distribution of PRC1 in the central spindle of anaphase cells.

### 3.5 Increased RITA normalizes the PRC1 staining area in the central spindle

If RITA depletion is responsible for an enlarged area of PRC1 staining, increased RITA should normalize this staining area. To examine this issue, HeLa CRISPRa sgcon and HeLa CRISPRa sgRITA cells with enhanced RITA expression (Supplementary Figures S6A–C) were stained for α-tubulin, PRC1 and DNA for microscopy (Supplementary Figure S6D). Indeed, the evaluation revealed that the staining area of PRC1 was normal in HeLa CRISPRa sgRITA cells (Supplementary Figure S6E). In particular, like control HeLa CRISPRa sgcon cells, PRC1 accumulated in the center of the central spindle of HeLa CRISPRa sgRITA cells (Supplementary Figure S6D), possibly at the plus ends of MTs of the central spindle. This interesting result further links RITA to the distribution of PRC1 in the central spindle of anaphase cells.

### 3.6 Less organized MT bundles of the central spindle upon RITA reduction

**FIGURE 6:**
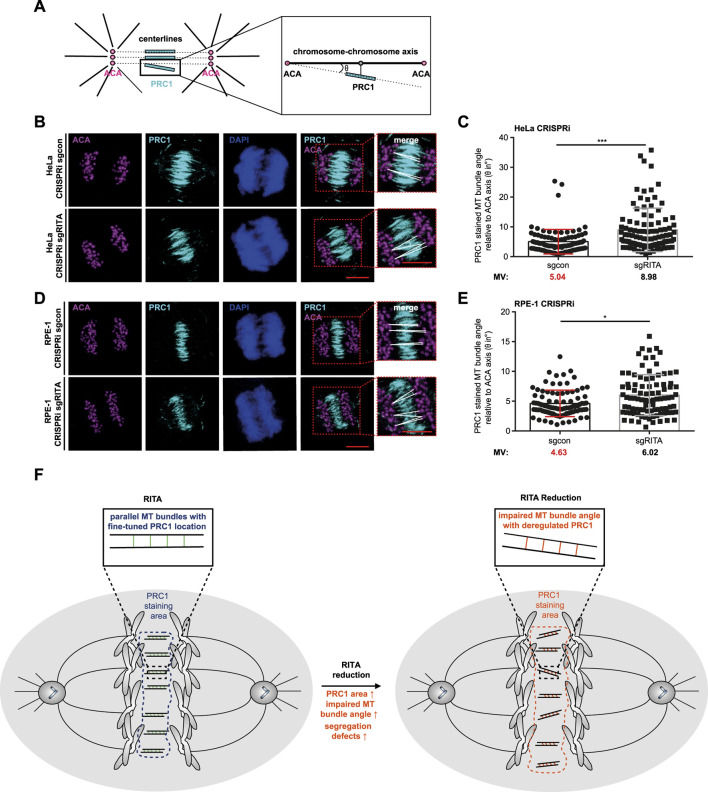
Increased MT bundle angles in the central spindle of cells depleted of RITA. **(A)** Schematic illustration of the angle measurement of PRC1 stained MT bundles. The angles (θ) of PRC1 stained MT bundles (cyan lines) were measured relative to centerlines (black dotted lines) connecting the staining of paired centromeres using ACA, a centromere/kinetochore marker (magenta circles), detailed by a zoomed region (black line box). Black lines represent spindle pole MTs. **(B)** HeLa CRISPRi sgRITA cells and control cells were stained for PRC1 (cyan), ACA (magenta) and DNA (DAPI, blue) for angle measurements. Representatives are shown. Scale: 10 µm. Insets depict magnified measured region. Scale: 10 µm. White lines indicate the measured angle (θ). **(C)** The angles of MT bundles stained with PRC1 were evaluated, relative to the centerlines connecting paired staining signals of ACA. The results are from three independent experiments (n = 3, 90 angles from at least 30 anaphase cells for each condition in each experiment) and presented as bar scatter blots, mean ± SD. Mann-Whitney U test was performed. ***p < 0.001. **(D)** hTERT RPE-1 CRISPRi sgRITA cells and their control cells were stained as indicated for angle measurements. Representatives are shown. Scale: 10 µm. Insets depict magnified measured region. Scale: 10 µm. White lines indicate measured angles (θ). **(E)** The angles of MT bundles stained with PRC1 were evaluated, relative to the centerlines connecting paired staining signals of ACA. The results are from three independent experiments (n = 3, 90 angles from at least 30 anaphase cells for each condition in each experiment) and presented as bar scatter blots, mean ± SD. Mann-Whitney U test was performed. ***p < 0.001. **(F)** A schematic illustration showing that RITA is involved in the distribution of PRC1 in the central spindle. Its reduction enlarges the staining area of PRC1, increases the MT bundle angles relative to paired ACA and affects the formation of the central spindle, which may contribute to defective chromosome segregation.

PRC1-crosslinked MTs initially form loose arrays, which become rearranged into bundles during anaphase, important for chromosome segregation (Matkovic et al., 2022; Do Rosario et al., 2023). Cells depleted of RITA often displayed fewer parallel MT bundles (Figures 5A, D), pointing to an improper organization of MT bundles in the central spindle. To characterize this observation in more detail, we employed an angle evaluation of MT bundles (Carlini et al., 2022). HeLa CRISPRi sgRITA and control cells were stained for PRC1, ACA and DNA, and confocal images were taken from early anaphase cells (Figure 6B). The angles of individual PRC1-stained MT bundles in the central spindle were quantified, relative to the axis lined by paired ACA staining (Figure 6A). In fact, the angles were increased in HeLa CRISPRi sgRITA cells compared to HeLa CRISPRi sgcon cells (Figure 6C). This observation was further corroborated with hTERT RPE-1 CRISPRi sgRITA cells (Figures 6D, E). The data suggest that MT bundles are not properly rearranged in the central spindle upon the depletion of RITA, which could affect chromosome segregation in anaphase cells.

## 4 Discussion

In the present work, we show that RITA partially co-localizes with PRC1 throughout mitosis, particularly in the central region of the central spindle in anaphase and in the midbody during cytokinesis. Stable as well as transient reduction of RITA in tumor cell lines, normal cells or MEFs results in an enlarged area of PRC1 staining in mitotic spindles of prometaphase and metaphase cells, and in the central spindle of anaphase cells as well. Moreover, the central spindle shows often unorganized MT bundles in diverse cell lines upon suppression of RITA. In contrast, HeLa cells stably overexpressing RITA showed normal staining area of PRC1 and display well-organized MT bundles of the central spindle. These findings indicate that RITA may be involved in the distribution of the important central spindle regulator PRC1 during mitosis.

The organization and function of the central spindle are spatiotemporally controlled by a variety of regulators, including kinesin motors, non-motor MAPs and kinases (Wadsworth, 2021). Being a MAP, RITA is an important modulator of MT stability and dynamics (Steinhauser et al., 2017). Here we present an additional role of RITA in the spindle distribution of PRC1, a key regulator of the central spindle assembly by conducting MT bundling and crosslinking (She et al., 2019). MT binding of PRC1 is mediated by a structured domain with a spectrin-fold and an unstructured Lys/Arg-rich domain, which are connected by a linkage that forms well-defined cross-bridges between antiparallel filaments *in vitro* (Subramanian et al., 2010). However, it is not defined, which MT features impact its MT binding and bundling activity *in vivo*.

We show that FLAG-RITA precipitates PRC1 as well as tubulin. Further assays using purified RITA and PRC1 proteins are required to study if RITA directly interacts with PRC1. This could also be that RITA and PRC1 form independent, non-mutually exclusive complexes with tubulin, as both RITA and PRC1 are tubulin-binding proteins (Mollinari et al., 2002; Steinhauser et al., 2017). Moreover, this may be an indirect association mediated by other factors, such as MT post-modifications. If fact, RITA coats the surface of MTs, interferes with MT properties *in vitro* as well as *in vivo*, and affects α-tubulin acetylation (Steinhauser et al., 2017). This post-translational modification of α-tubulin is associated with long-lived MTs (Shida et al., 2010) by altering the MT lattice structure and changing the interaction with MAPs (Bar et al., 2022). It is therefore conceivable that RITA covers spindle MT fibers that may modulate PRC1’s proper MT binding and distribution. Indeed, our data from CRISPRi and CRISPRa cells, RITA knockout and knockdown MEFs, and various cancer cell lines suggest that RITA may modulate PRC1 distribution in mitotic spindles in metaphase cells and in the central spindle of anaphase cells. Depletion of RITA may also affect PRC1’s activity to bundle/crosslink antiparallel MTs into higher ordered structural arrays, leading to its enlarged and non-structural distribution in the spindles. To support this notion, reduced RITA causes more non-parallel MT bundles with enlarged angles relative to corresponding separating chromosomes in the central spindle. Since the localization and activity of PRC1 are regulated by other MAPs, such as Kif4A, and mitotic kinases, including cyclin dependent kinase 1 (Cdk1), Plk1 and Aurora B (Mollinari et al., 2005; Zhu and Jiang, 2005; Hu et al., 2012; Nunes Bastos et al., 2013), further investigations are needed to precisely uncover whether these regulators are involved in altered distribution of PRC1 in the central spindle of anaphase cells depleted of RITA.

The central spindle MTs and chromosome movements are strongly coupled in anaphase (Yu et al., 2019), suggesting the importance of a proper spindle in separating chromosomes. Moreover, perturbation of MT bundles also causes inefficient correction of erroneous kinetochore-MT attachments in metaphase and leads to defective chromosome segregation in anaphase (Matkovic et al., 2022). We show here that the reduction of RITA decreases the inter-centromere distance and impairs the proper formation of MT bundles that may contribute to defective chromosome congression and segregation during mitosis, which was observed in HeLa and HCT116 cells transiently depleted of RITA, and RITA knockout MEFs (Steinhauser et al., 2017). In the present work, we further substantiate the observed mitotic phenotype showing impaired chromosome movements in HeLa CRISPRi sgRITA cells, hTERT RPE-1 CRISPRi sgRITA cells as well as U-2OS cells transiently depleted of RITA. These findings strongly suggest RITA’s general significance in guarding chromosome integrity in cell division. Increased chromosomal instability, due to reduced MT-kinetochore tension and impaired central spindle shown here, deregulated MT dynamics (Steinhauser et al., 2017) and abnormal Aurora A activity (Kreis et al., 2019), could be linked to malignant development.

Although the reduction of RITA results in mitotic defects in HeLa as well as hTERT RPE-1 cells, the cell proliferation rate of those cells, is relatively comparable to their control cells. It is well known that HeLa cells are p53 deficient (Matlashewski et al., 1986) that alters the cell response to mitotic defects, whereas hTERT RPE-1 cells are reported to be cGAS (cyclic GMP-AMP synthase) negative (Chen et al., 2020), which may affect the cellular outcome of RITA knockdown. Studies are needed to clarify the discrepancy between mitotic defects and the cell viability in these cells knocked down of RITA.

In conclusion, we show that RITA is involved in the spindle distribution of PRC1 during mitosis. Importantly, its reduction enlarges the staining area of PRC1 in prometaphase, metaphase as well as anaphase, and affects MT bundle formation in the central spindle, contributing to defective chromosome congression/segregation and cytokinesis defects (Figure 6F). Further investigations are required to disclose the function of RITA in the central spindle organization, in particular, the precise molecular mechanisms by which RITA modulates PRC1 and possibly its various partners in the central spindle.

## Data Availability

The original contributions presented in the study are included in the article/Supplementary Material, further inquiries can be directed to the corresponding author.

## Abbreviations

ACA, anti-centromere antibody; Cdk1, cyclin dependent kinase 1; cGAS, cyclic GMP-AMP synthase; CRISPR, clustered regularly interspaced short palindromic repeats; KIF4A, kinesin family member 4A; MTs, microtubules; MEFs, mouse embryo fibroblasts; Plk1, Polo-like kinase 1; PRC1, protein regulator of cytokinesis 1; RITA, RBP-J interacting and tubulin-associated protein.
