# Supplementary figures and images for: Involvement of RBP-J interacting and tubulin-associated protein in the distribution of protein regulator of cytokinesis 1 in mitotic spindles
Source: Front Cell Dev Biol. 2025 Jan 7;12:1472340. doi: 10.3389/fcell.2024.1472340 (PMC11747798; doi:10.3389/fcell.2024.1472340)

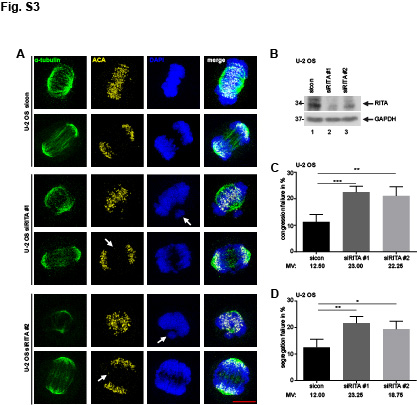

Supplement: Supplementary file 1 [file Image3.jpeg]

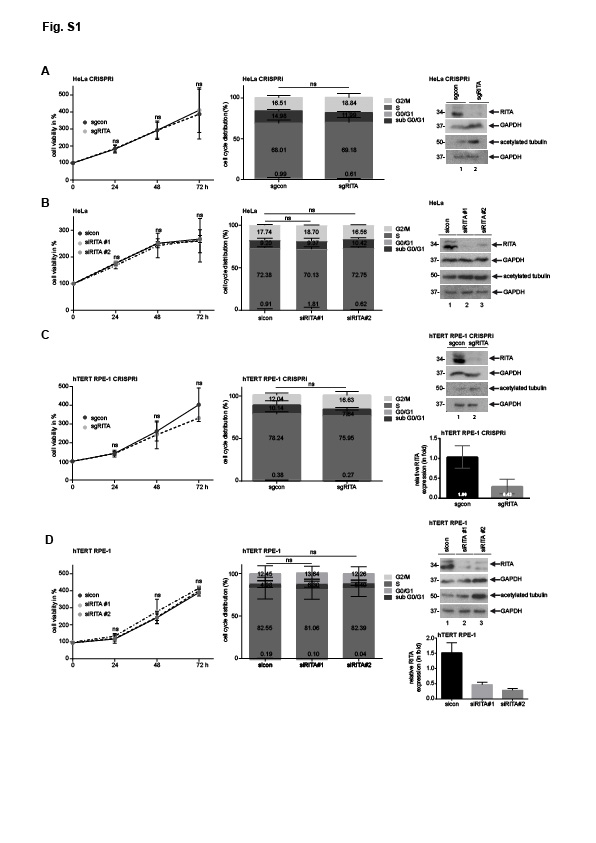

Supplement: Supplementary file 2 [file Image1.jpeg]

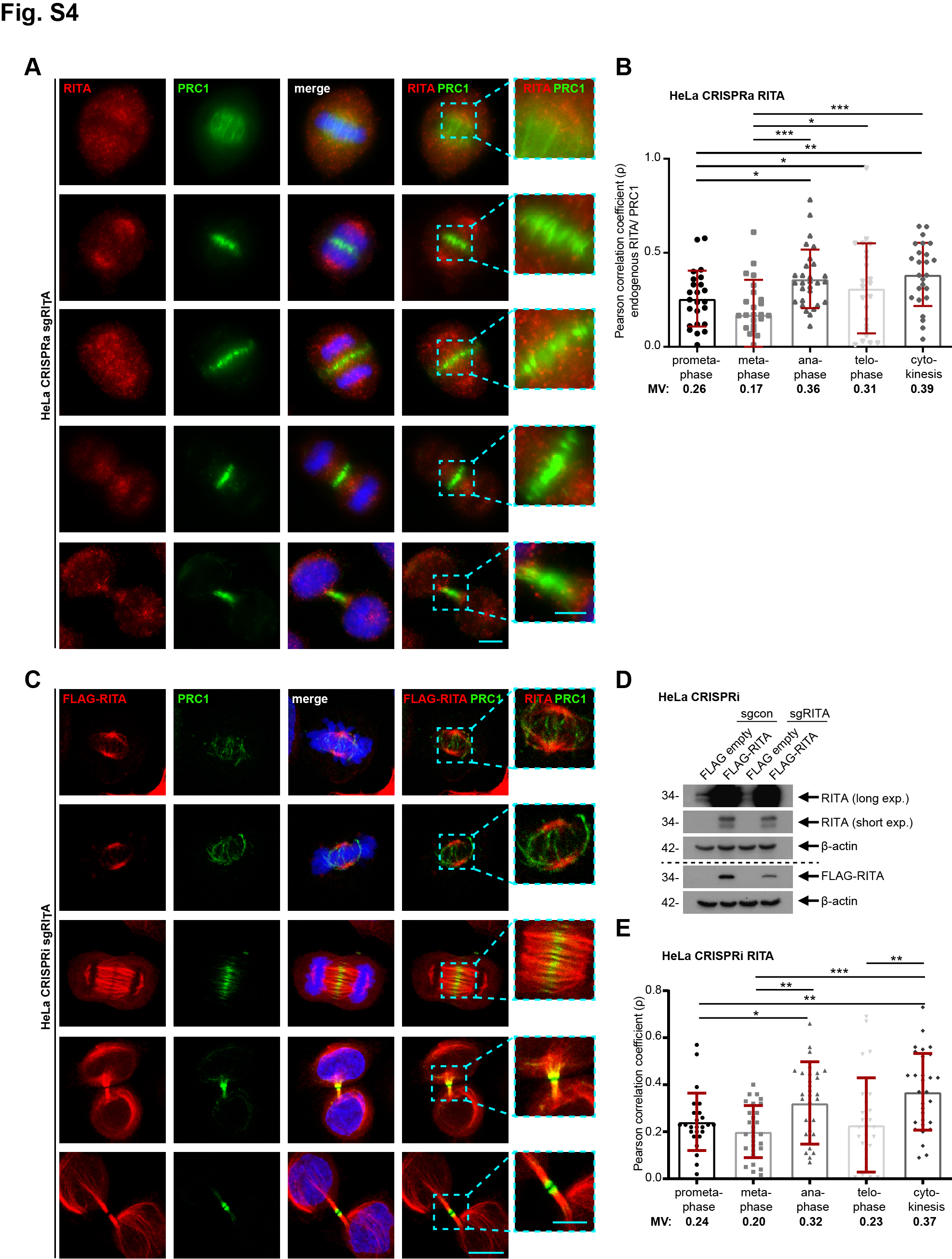

Supplement: Supplementary file 3 [file Image4.jpeg]

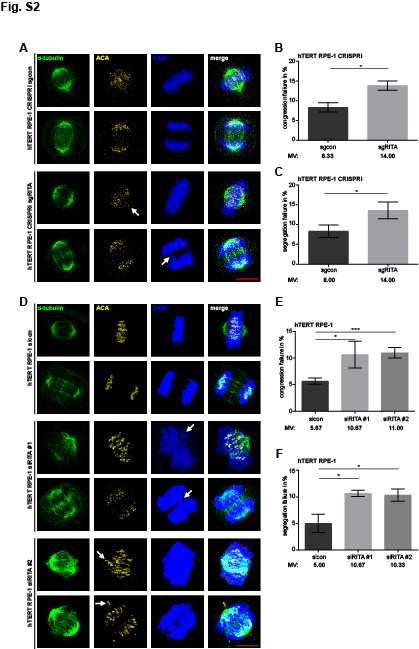

Supplement: Supplementary file 4 [file Image2.jpeg]

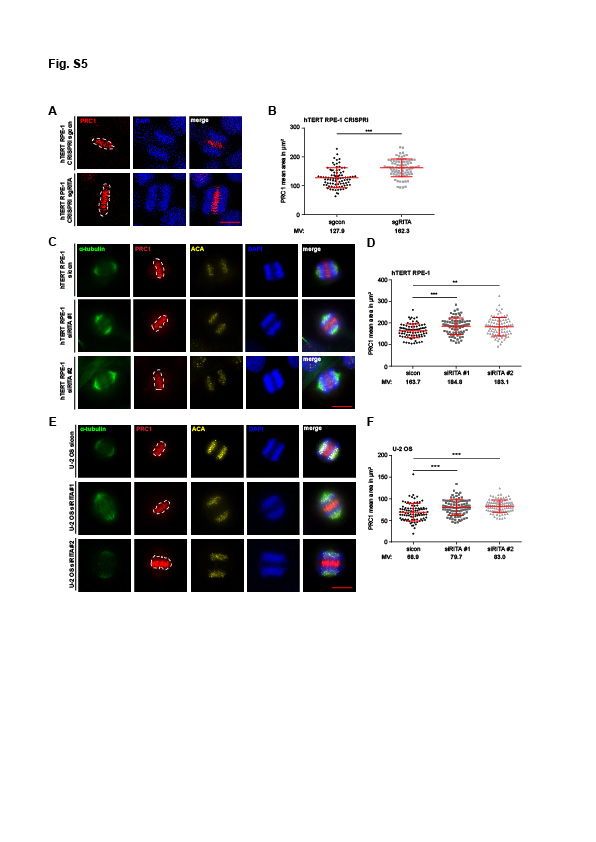

Supplement: Supplementary file 5 [file Image5.jpeg]

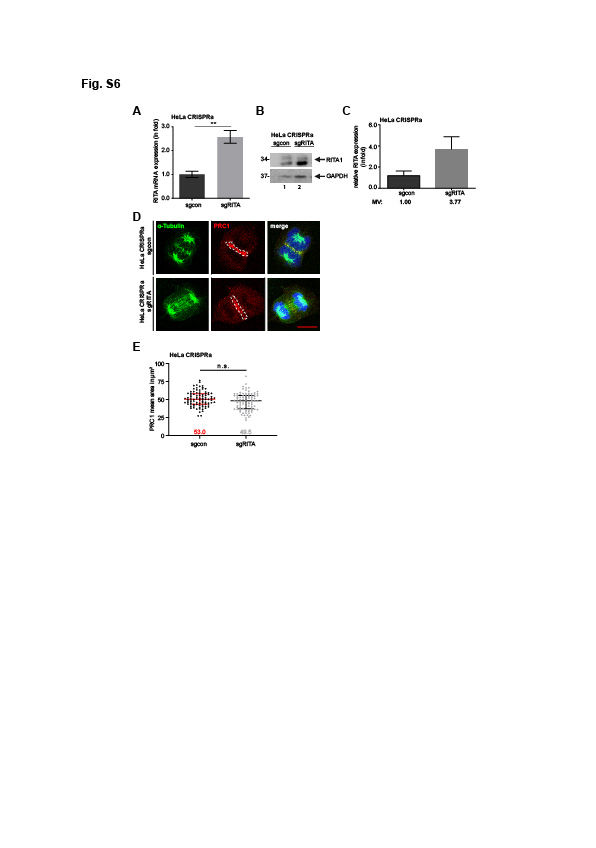

Supplement: Supplementary file 6 [file Image6.jpeg]
